# Alcohol-based mouthwash as a risk factor of oral cancer: A systematic review

**DOI:** 10.4317/medoral.23085

**Published:** 2019-10-27

**Authors:** Marina Ustrell-Borràs, Bassel Traboulsi-Garet, Cosme Gay-Escoda

**Affiliations:** 1 DDS. Oral Surgery Master of the EFHRE International University, Barcelona, Spain; 2 DDS. Fellow of Master’s Degree Program in Oral Surgery and Implantology, Faculty of Medicine and Health Sciences, University of Barcelona, Barcelona, Spain; 3 MD, DDS, MSc, PhD, EBOS, OMFS. Chairman and Professor of Oral and Maxillofacial Surgery, Faculty of Medicine and Health Sciences, University of Barcelona, Barcelona, Spain. Director of Master’s Degree Program in Oral Surgery and Implantology (EFHRE International University/FUCSO, Belize City, Belize). Coordinator/Researcher of the IDIBELL Institute. Head of Oral Surgery, Implantology and Maxillofacial Surgery Department of the Teknon Medical Center, Barcelona, Spain

## Abstract

**Background:**

Oral and pharynx cancer represent a serious global problem, reaching an incidence of half a million cases annually. The role of tobacco and alcohol have been studied and proven to be one of its risk factors. We also know that mouthwashes contain a variable percentage of alcohol, so there is a reasonable concern about their role in carcinogenesis.

**Material and Methods:**

To answer the PICOS (Population; Intervention; Comparison; Outcomes; Study) question: "Do patients (Population) who use alcohol-based mouthwashes (Intervention) compared to those who do not use them (Comparison) have higher acetaldehyde levels in saliva or higher risk of oral cancer development? (Outcomes)" Meta-analyses, systematic reviews, randomized and non-randomized clinical trials, case-control studies, and prospective and retrospective cohort studies were included (Study). Two independent authors conducted literature screening through MEDLINE, Scopus and the Cochrane Library, and they also conducted article and data extraction to undertake quality analyses. The main outcome measures were salivary acetaldehyde levels or the risk of oral cancer development. The most relevant data was extracted and the risk of bias from the studies included was also evaluated.

**Results:**

Out of 497 potentially eligible papers, 8 studies were included in the qualitative analysis which include a total of 43,499 subjects: two meta-analyses, a clinical trial, three case-control studies and two cohort studies. One study (n = 3,926) found a relationship between alcohol mouthwash and oral cancer, two studies (n = 25,033) found this relationship when a high frequency of mouthwash was present, three studies (n = 14,482) failed to find this relationship and 2 studies (n = 58) found a temporary increase of acetaldehyde levels in saliva after alcohol mouthwash.

**Conclusions:**

It cannot be guaranteed that the use of mouthwash represents an independent risk factor for the development of head and neck cancer. However, the risk does increase when it occurs in association with other carcinogenic risk factors.

** Key words:**Oral Cancer, Oropharyngeal Neoplasms, Mouthwashes, Mouth rinse, Ethanol, Acetaldehyde, alcohol.

## Introduction

There is an estimated incidence of oral and pharynx cancer of half a million per year resulting in 250.000 deaths annually in United States.

Most of them are squamous cell carcinoma of the oral cavity (SCC). The main risk factors for head and neck cancer are tobacco and alcohol; the risk increases when both factors are present. Approximately 70% of cancers in this region could be explained by exposure to one or both factors (86% in the oral cavity and 86% in the larynx). Currently, poor oral hygiene and chronic infection from Human Papillomavirus (HPV) could be added to the main risk factors of oropharyngeal cancer, highlighted in young patients (3,4).

This risk from alcohol consumption increases ten times in heavy drinkers compared to abstainers or irregular drinkers (5). The total volume of ethanol in alcoholic beverages seems to be one of the main determinants of risk, so a dose-dependent relationship is observed.

Although there is a short period of exposure time to alcohol, this seems to increase the permeability of the oral mucosa to potential carcinogens, as well as, specific Nitrosamines of tobacco (5).

Many mouth rinses contain alcoholic concentrations between 5 - 27%. This is usually used as a solubilizer, stabilizer, preservative, anti-plaque efficacy enhancer and as a way to obtain a distinctive flavor. On the other hand, in a similar way to the alcoholic solutions ingested, mouthwashes with alcoholic concentrations between 18 - 27% also potentiate the effect of the essential oils to achieve a high penetration in soft tissues (in 30 seconds) (5).

Some biochemical and epidemiological studies suggested that acetaldehyde, the first metabolite of ethanol, plays an important role in the carcinogenesis of alcohol in oral, pharynx, larynx and esophagus cancer (the upper respiratory tract) (6,7). This metabolite can be seen to increase in the human body, especially in the salivary medium, after consuming alcoholic beverages (6). This increase could possibly occur with alcohol-based mouth rinses, and their use could be a possible risk factor for oral and pharynx cancer.

The mechanism for the acetaldehyde production of ethanol can come from the metabolism of ethanol itself through oral microflora or conversion by the epithelial cells. It has been shown that poor oral hygiene increases the production of acetaldehyde, in addition to other factors (smoking and heavy drinking), producing a carcinogenic potential in the oral cavity and upper airways (6,7).

During the last thirty years, there has been an attempt to find a possible association between the use of mouthwash with alcohol and its relationship with oral cancer. However, epidemiologically, there has been no conclusive evidence. Few epidemiological studies are found in the literature and they have contradictory results (5).

Many of the commercially available mouth rinses contain considerable amounts of ethanol. Most of the adult population use this type of mouth rinse, so it could be transcendent in public health to certify its possible carcinogenic potential.

Gandini *et al*. (5) published a meta-analysis in which they tried to reveal whether there was or not a real relationship with alcoholic mouth rinses and a higher rate of oral, or pharynx and/or larynx cancer. However, they did not use any indicator except for the presence of cancer as a result variable, which was not very prevalent or may not have already appeared at the moment of the study.

There seems to be evidence of the relationship between the consumption of mouth rinse with an alcoholic solution and the production of acetaldehyde. The literature also suggests that there is a clear relationship between the metabolite of ethanol and oral and upper airway cancer (6,7).

Therefore, the increase of salivary acetaldehyde can be an indicator of risk in oral cancer, which allows assessing other parameters such as the duration of the metabolite in the mouth after using mouth rinse or if the alcoholic concentration increases the risk of cancer. The aim of this study was to determine the relationship between the use of alcohol-based mouthwashes and salivary acetaldehyde levels and the risk of oral cancer development.

## Material and Methods

The present article follows the Preferred Reporting Items for Systematic Reviews and Meta- Analyses declaration (PRISMA) (8). A meta-analysis would have been performed if the studies included had been carried out under homogeneous conditions.

- Selection criteria

Inclusion criteria were based on the following PICOS question: patients with no age, gender or medical condition restrictions (Patient); alcohol-based mouthwashes use (Intervention); no mouthwashes use (Comparison); oral cancer or the acetaldehyde levels in saliva (Outcome). Meta-analyses, systematic reviews, randomized and non-randomized clinical trials, case-control studies and prospective and retrospective cohort studies were included. Just articles published in the last 10 years were included and no language restrictions were applied.

Animal studies, case series, case reports and technical notes were excluded. Studies of digestive tract cancer not including the oral area or based on alcohol sugars were also excluded.

The main outcome variable was the level of acetaldehyde in saliva. The secondary outcome variable was the risk of oral cancer development.

- Search strategies

An electronic search in Pubmed (MEDLINE), Scopus and the Cochrane Library (Central) databases was conducted until April 13th, 2018. Even though the search was initially limited to the last 10 years, the first studies began in 2006, so in the end, articles published between 2006 and April 13th, 2018 were included.

The search strategy was: ((“mouth rinse” [MH] AND “acetaldehyde” [MH]) OR (“mouth rinse” [MH] AND “alcohol” [MH])) OR (“Acetaldehyde” [MH] AND (oral cancer* [TIAB] OR tobacco* [TIAB] OR oral bacteria* [TIAB])) OR (“mouthwashes” [MH] AND (“acetaldehyde” [MH] OR “ethanol” [MH])) OR (“acetaldehyde” [MH] AND alcohol* [TIAB] AND (“mouth rinse” [MH] OR “mouthwash” [MH])) OR (“acetaldehyde” [MH] AND (cancer* [TIAB] OR “tobacco” [MH] OR (oral* [TIAB] AND bacteria* [TIAB]))) OR (“acetaldehyde” [MH] AND alcohol* [TIAB] AND oral*[TIAB]).

- Study selection and data selection process

**Figure 1:**
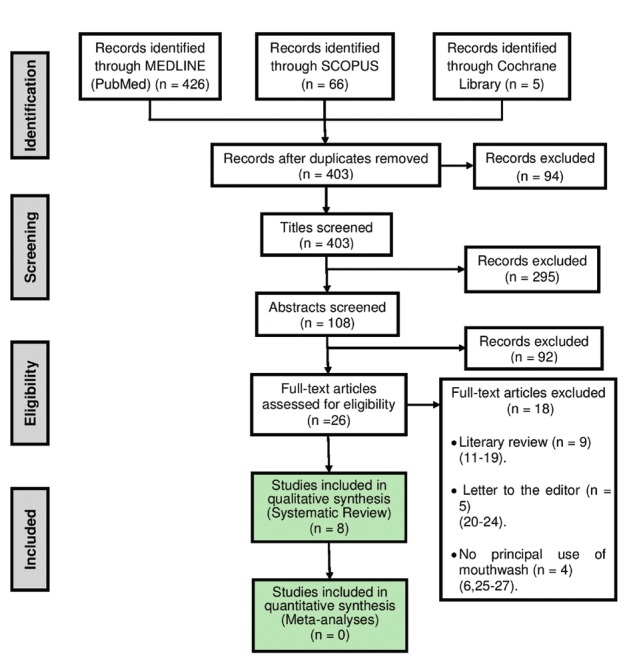
Flowchart illustrating study selection process.

After eliminating duplicate studies, two independent examiners (MUB, BTG) selected the studies by first screening the titles and abstracts of the articles to determine their eligibility. The full text of the selected articles were then evaluated according to selection criteria. Any discrepancies were resolved by a third reviewer (CGE). The excluded articles and the reasons for their exclusion were recorded in this final stage (Fig. 1). Two Cohen’s kappa were calculated to determine the interrater reliability for both selection stages between both examiners (MUB, BTG).

- Data extraction

The data was extracted from the articles selected by one of the authors (MUB). After analyzing all the selected articles, the following data were identified: 1) the author or authors, 2) year of publication, 3) country of origin, 4) design of the study, 6) details of the participants, 7) intervention, 8) comparison, 9) outcomes and 10) follow-up. If the included studies had a homogeneous methodology, a meta-analysis of the data would be possible.

- Risk of bias in individual studies

As part of the data extraction process, two reviewers (MUB, BTG) independently assessed the risk of bias in the included studies.

For cohorts and case-control studies, the risk of bias was assessed according to the Newcastle-Ottawa Scale (NOS) (9) (Table 1). A quantitative method was used to compare the quality level between different studies, giving one point for each satisfactory response of the items evaluated. Studies with a NOS level ≥ 6 were considered as high quality studies with a low risk of bias. Those with a NOS level < 6 points were considered as low quality studies with a high risk of bias (9).

For clinical trial or meta-analysis studies, the quality evaluation system was SIGN (10), a categorization system that grants a level of evidence by evaluating the type of design and the execution. A Table with the data of quality evaluation and risk of bias was made (Table 1).

**Table 1:**
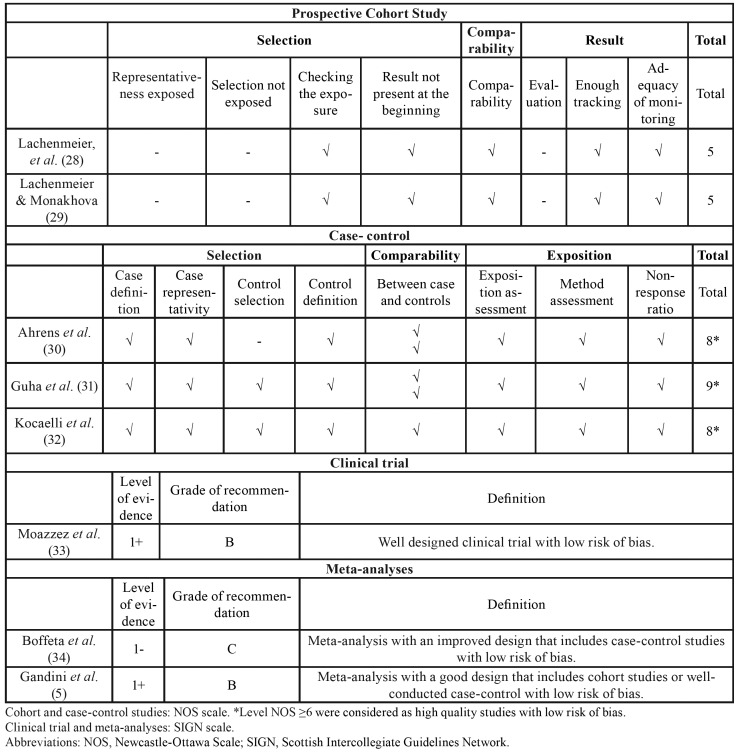
Quality assessment of included studies.

## Results

- Study selection and description

The flow chart for the selected articles used in this systematic review can be seen in Fig. 1. 497 references were obtained in the initial search. After the duplicates were eliminated, and the titles and abstracts of the articles were evaluated, a total of 26 articles were chosen to analyze the full text, in which the Cohen’s kappa value was 0.982, so the interrater reliability was excellent.

18 articles were excluded according to the selection criteria. The exclusion reasons were: literary reviews (11-18), letters to the editor (20-24) and use of mouthwash with no alcoholic base (6,25-27). The Cohen’s kappa value of this final eligibility stage was 0.92, so the interrater reliability was also good.

Finally, 8 studies were included in the qualitative analysis (Table 1) (5,28-34). Of the studies included, there were two meta-analyses (5,34), a clinical trial (33), three case-control studies (30-32) and two cohort studies (28,29) (Fig. 1).

- Risk of bias assessment

As shown in Table 1, the two cohort studies (28,29) obtained the same score: 4 out of 9, with a high risk of bias. Two case-control studies obtained a score of 9 points (31,32), and the other one obtained a score of 8 points (30), all of them with a low risk of bias.

Both clinical trial (33) and one of the meta-analysis (5) obtained a score of 1+ with a low risk of bias in the SIGN classification. The other meta-analysis obtained a score of 1- in the same classification, with a high risk of bias (34).

- Data extraction: qualitative synthesis

Among all the studies, a total of 43,499 subjects were included. In one of the meta-analysis (n = 14,276) (5) the number of men and women was not reported. The remaining subjects ((n = 29,223)

) of the other included articles in the present study, 21,754 were male and 7,469 were female. The age ranged between 22 and 75 years old. The descriptive variables of the included studies can be found in Table 2.

**Table 2:**
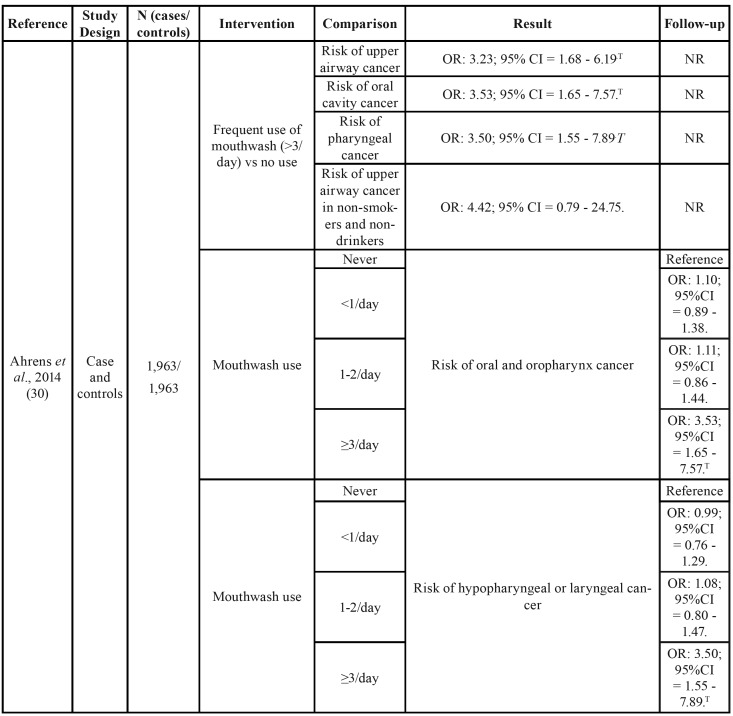
Summary of the characteristics of the studies included in the present systematic review.

**Table 2 cont.**
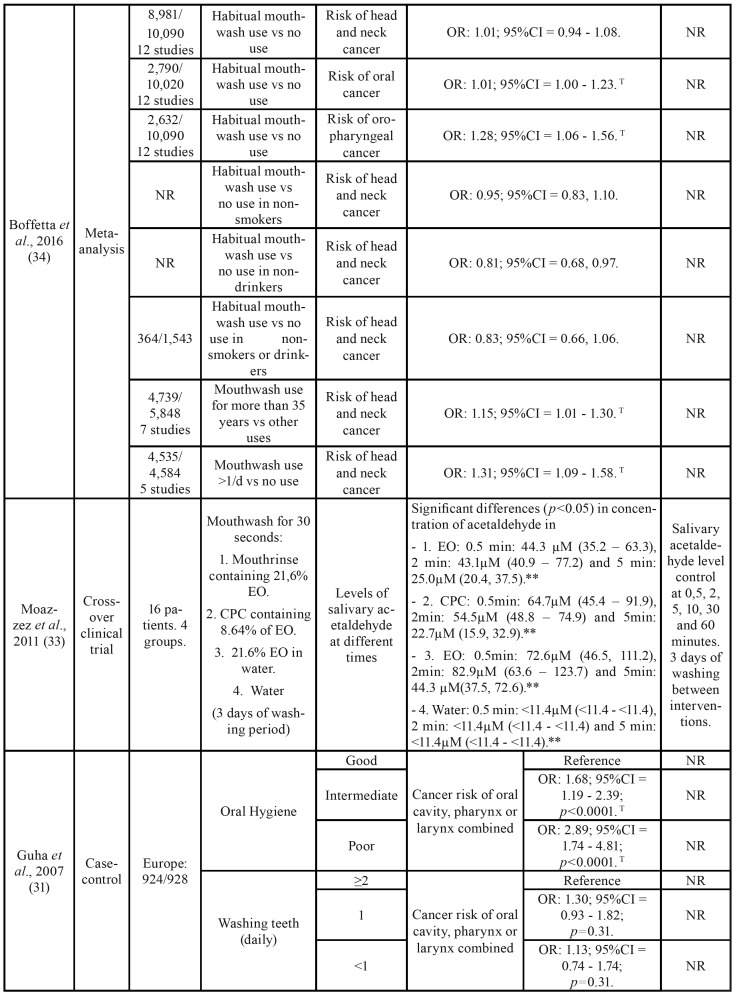
Summary of the characteristics of the studies included in the present systematic review.

**Table 2 cont.**
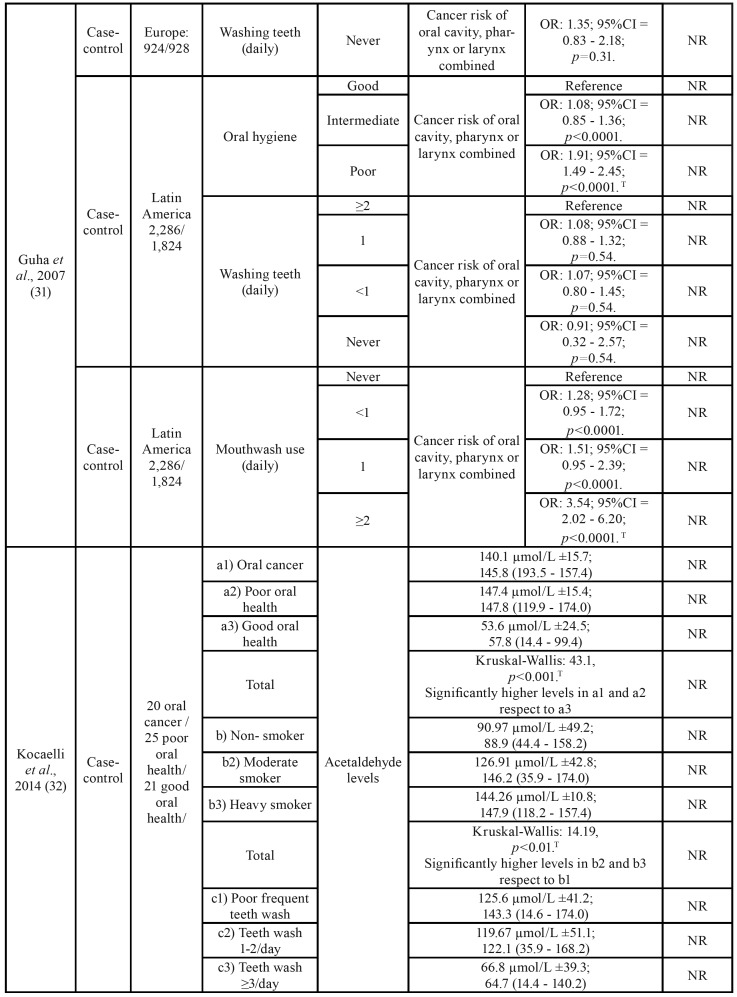
Summary of the characteristics of the studies included in the present systematic review.

**Table 2 cont.**
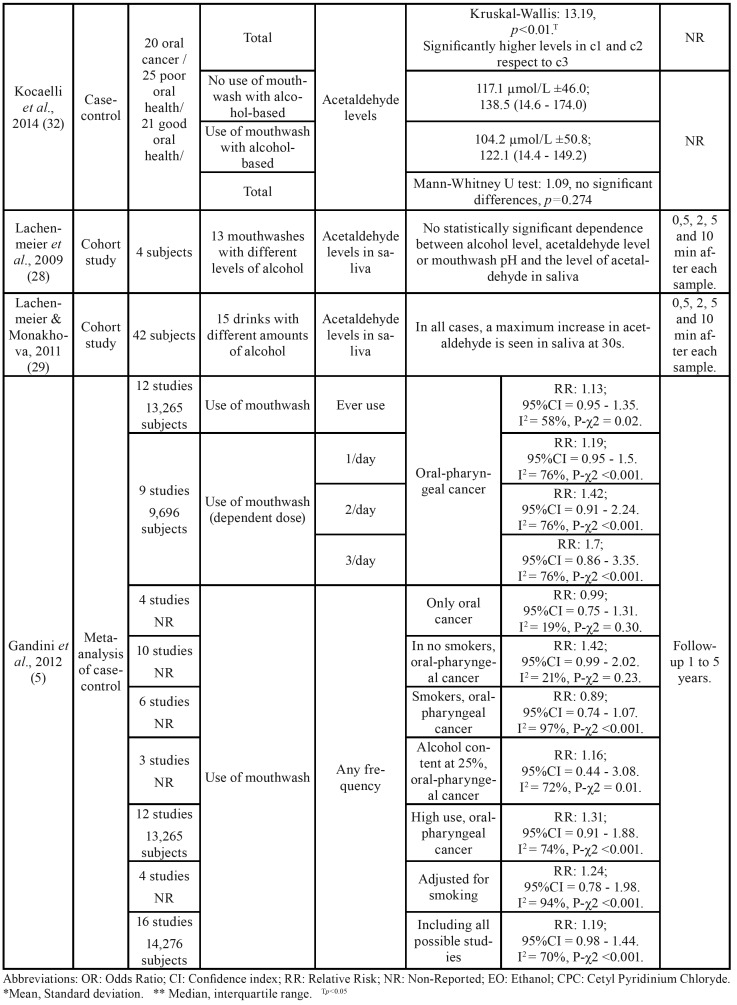
Summary of the characteristics of the studies included in the present systematic review.

Ahrens *et al*. (30) found an increased risk of upper airway cancer with the use of alcoholic mouthwash ≥3 times/day as opposed to not using it (OR: 3.23; 95%CI = 1.68 - 6.19). This effect was greater if it was restricted to oral cavity cancer (OR: 3.53; 95%CI = 1.65 - 7.57) and pharynx cancer (OR: 3.50; 95%CI = 1.55 - 7.89) (Table 2). After an adjustment for different confounding factors, a significantly increased risk of head and neck cancer was obtained in patients who used mouthwash three times/day or even more when it was adjusted for age, sex and study center (OR: 2.31; 95%CI = 1.27 - 4.21); also in smokers and drinkers (OR: 2.91; 95%CI = 1.52 - 5.57), educational level (OR: 3.01; 95%CI = 1.56 - 5.79) and for consumption of fruits and vegeTables (OR: 3.23; 95%CI = 1.68 - 6.19).

Boffetta *et al*. (35) did not observe differences between the habitual use of mouthwash compared to the non-use of alcohol-based mouthwash, without considering the frequency per day, for the risk of head and neck cancer (OR: 1.01; 95%CI = 0.94 - 1.08). However, there were statistically significant differences when it came to oral and oropharynx cancer, but with a discrete effect (OR: 1.01; 95%CI = 1.00 - 1.23 and OR: 1.28; 95%CI = 1.06 - 1.56 respectively).

Subjects who had used the mouthwash longer (>35 years) also had an increased risk of upper airway cancer (OR: 1.15; 95%CI = 1.01 - 1.30), as well as those using it more than once per day (OR: 1.31; 95%CI = 1.09 - 1.58).

As in the previous two studies, Guha N. *et al*. (31) confirmed the positive relationship between mouthwash use and cancer (oral, pharynx or larynx cancer). Specifically, using subjects who did not use mouthwash as a reference, a higher risk of cancer was found for subjects who used it ≥2 times a day (OR: 3.54; 95%CI = 2.02 - 6.20; *p*<0.0001) and although it is a positive OR, there was no significant difference if the use of mouthwash was once a day (OR: 1.51; 95%CI = 0.95 - 2.39; *p*<0.0001) or less than 1 use/day (OR:1.28; 95%CI = 0.95 - 1.72; *p*<0.0001). All this data was obtained in centers located in Latin America (Table 2).

Both Europe or Latin America samples had a significantly higher risk of oral, pharynx and larynx cancer in the group of poor oral hygiene versus the group of good oral hygiene (OR: 2.89; 95%CI = 1.74 - 4.81; *p*<0.0001 for the European sample, and OR: 1.91; 95%CI = 1.49 - 2.45; *p*<0.0001 for the American sample). However, in both cases no differences were found between the group that brushes their teeth ≥2 times/day and the group that never brushes it (OR: 1.35; 95%CI = 0.83 - 2.18; *p*=0.31 for the European sample and OR: 0.91; 95%CI = 0.32 – 2.57; *p*=0.54 for the Latin American sample). These results were obtained when patients were analyzed without considering possible confounding factors such as smoking and alcohol. The tobacco and alcohol habit modified the risk of cancer according to the use of mouthwash (31).

Kocaelli *et al*. (32) studied the levels of salivary acetaldehyde in different groups of subjects. They found significantly higher levels of acetaldehyde in subjects with oral cancer (a1) and poor oral health (a2) than those with good oral health (a3) (Kruskal-Wallis: 43.1; *p*<0.001); no significant differences were found between subjects with oral cancer and poor oral health (Table 2). The acetaldehyde levels in saliva were significantly higher in subjects who occasionally washed their teeth (c1) and in subjects with 1-2 washes/day (c2) than in subjects with ≥3 daily washes (c3) (Kruskal-Wallis: 13.19, *p*<0.01), but no differences were found between occasional tooth washing and 1-2 washes/day (Table 2). Finally, no differences were found in the levels of acetaldehyde in saliva between subjects who used mouthwash daily and those who did not use it (104.2 μmol/L ±50.8 and 117.1 μmol/L ±46.0 respectively). (Mann-Whitney U test: 1.09; *p*=0.274).

Other studies that analyzed acetaldehyde levels were Lachenmeier *et al*. (28) and Lachenmeier & Monakhova (29). In the first one, acetaldehyde levels were studied in 4 healthy subjects after using 13 mouthwashes with different alcohol concentrations for 30 seconds. Alcohol concentrations were the following: 6,8%, 6,9%, 9,4%, 10,0%, 10,0%, 10,9% 20,0%, 21,8%, 21,8%, 22,0%, 22,2%, 22,3%, 26,8%. Samples were taken at 0.5, 2, 5 and 10 minutes later. No relationship was found between different alcohol concentrations, acetaldehyde levels or pH. Lachenmeier & Monakhova (29) took the samples with the same time interval than the previous study, but in this paper there were 15 alcoholic beverages with the following concentrations: 5%, 5,5%, 13%, two of 15%, 16%, six of 40%, two of 41%, and 43%. Acetaldehyde levels were compared in 42 subjects, and in all the cases, a maximum increase in acetaldehyde was observed in saliva at 30s.

According to Moazzez *et al*. (33), the levels of acetaldehyde in saliva were related to a time variable. They used 4 different groups, with different mouthwashes: I) mouth rinse containing 21.6% ethanol (Listerine Coolmint; Johnson& Johnson, Maidenhead, UK), II) Cetyl Pyridinium Chloride (CPC) containing 8.64% of ethanol, III) 21.6% of ethanol in water and IV) water as a control. Samples were taken after mouthwash at 30 seconds, 2, 5, 10, 30 and 60 minutes. In this study, a significant increase of acetaldehyde concentration in saliva was observed in the three samples of mouthwash with ethanol at 30 seconds, 2 and 5 minutes, with a subsequent decrease. The maximum peak was between 30 seconds and 2 minutes. On the other hand, Gandini *et al*. (5) did not find differences in oral or pharyngeal cancer according to mouthwash use (OR: 1.13; 95%CI = 0.95 - 1.35). No differences were found between mouthwash use and non-use in terms of risk for oral cancer (RR: 0.99, 95%CI = 0.75 - 1.31, I2 = 19%, P-χ2 = 0.30).

The relative risk summary estimates for 1-3 times a day of mouthwash showed a dose dependent trend but with no statistically significant increased risk for oral cancer, compared to no exposure: 1.19 (95%CI = 0.95 – 1.5), 1.42 (95%CI = 0.91 – 2.24) and 1.7 (95%CI = 0.86 – 3.35), respectively, with I2 = 76% and Chy-square *p*<0.001.

- Analysis according to smoking or alcohol habits

In the sample from Latin America in this last study, an increasing trend was observed according to the use of smoking as a modifying variable, increasing the risk of cancer. In non-smoking patients, the use of mouthwash ≥2 times/day compared to not using it obtained an OR: 2.71 (95%CI = 0.74 - 9.97; *p*=0.06) with no statistically significant differences. On the other hand, in ex-smoker patients an OR: 4.98 (95%CI = 1.72 - 14.43; *p*=0.003) and in smokers an OR: 9.15 (95%CI =2.13 - 39.22; *p*=0.0002), both with statistically significant differences. The differences were not significant when the use of mouthwash was 1 time/day (OR: 1.89 (95%CI = 0.45 - 7.87, *p*=0.06); OR: 1.94 (95%CI = 0.88 – 4.25, *p*=0.003); OR: 1.53 (95%CI = 0.76 – 3.08, *p*=0.0002) respectively), obtaining a positive dose-dependent trend (31).

In the sample of European subjects, in non-smoker and ex-smoker cases, no statistically significant differences in the risk of cancer were observed between subjects with poor and good oral hygiene (OR: 2.46 (95%CI = 0.25 - 23.94; *p*=0.36) and OR: 1.25 (95%CI = 0.32 - 4.84; *p*=0.78) respectively). Smokers with poor oral hygiene obtained an increased risk for oral, pharynx and larynx cancer compared to those smokers with good oral hygiene, with an OR: 3.60 (95%CI = 1.95 - 6.62; *p*<0.0001) (31).

In the sample from Latin America, considering the previous comparisons, in smokers and ex-smokers no differences were observed, with an OR: 1.74 (95%CI = 0.81 - 3.72; *p*=0.23) and OR: 1.20 (95%CI = 0.75 - 1.92; *p*=0.41) respectively; however they were observed in smokers (OR: 1.98, 95%CI = 1.37 - 2.85; *p*<0.0001) (31).

Ahrens *et al*. (30) found an increasing trend of risk but without statistical significance in non-smokers and non-drinkers, with a wide interquartile range (OR: 4.42; 95% CI = 0.79 - 24.75), while they were significant when no stratifying for smokers or drinkers was analyzed (OR: 3.23; 95%CI = 1.68 - 6.19), both using mouthwash ≥3 times/day.

Similarly, Boffetta *et al*. (35) found a statistically significant increased risk of cancer in the oral cavity and oropharynx when using mouthwash (OR: 1.01; 95%CI = 1.00 - 1.23 and OR: 1.28; 95%CI =1.06 - 1.56 respectively). However, by restricting the results to non-smokers, the risk was not statistically significant (OR: 0.95; 95%CI = 0.83 - 1.10); as well as in non-drinkers, who had statistically significantly less risk of cancer than those who used mouthwash (OR: 0.81; 95%CI = 0.68 - 0.97). In non-smokers or drinkers, the use of mouthwash did not increase the risk of head and neck cancer (OR: 0.83; 95%CI = 0.66 - 1.06).

Kocaelli *et al*. (32) found increased acetaldehyde levels in the group of heavy smokers (b3 in Table 2) or moderate smokers (b2) compared to non-smokers (b1) (Kruskal-Wallis: 14.19, *p*<0.01). However, no significant differences were found between heavy smokers and moderate smokers (Table 2).

Guha *et al*. (31) obtained a higher risk using mouthwash ≥2 times/day than not using it, whether they were alcohol or non-alcohol consumers (OR: 5.12; 95%CI = 2.20 - 11.92; *p*=0.0002 and OR: 4.96; 95%CI = 1.85 - 13.31; *p*=0.001 respectively). Oral hygiene was not assessed in the European case due to a limited sample. In the American sample, there were no significant differences in drinkers who presented good or bad oral hygiene (OR: 1.59; 95%CI = 0.86 -2.94; *p*=0.12), but they were significant in drinkers, with a higher risk for subjects with poor oral hygiene than those with good oral hygiene (OR: 1.81; 95%CI = 1.36 - 2.40; *p*<0.0001). In this case, there were no significant differences between the number of tooth washes per day and the consumption of alcohol in terms of the risk of oral, pharynx or larynx cancer (31).

Gandini *et al*. (5) did not find differences in oral or pharyngeal cancer according to mouthwash use in the non-smoker sample (RR: 1.42; 95%CI = 0.99 - 2.02, I2 = 21%, P-χ2 = 0.23), smokers (RR: 0.89; 95%CI = 0.74 - 1.07, I2 = 97%, P-χ2<0.001) or drinkers of beverages with an alcoholic content of 25% (RR: 1.16; 95%CI = 0.44 – 3.08, I2 = 72%, P-χ2 = 0.01).

## Discussion

During the last few decades it has been controversial if the use of alcohol-based rinsing increases the risk of oral cancer, oropharynx or other head and neck cancers. Although there were already published studies on the subject (5,33,34), there was no consensus on whether it was a risk factor for cancer.

There is evidence that acetaldehyde is a carcinogenic substance (6,36,37), and there seems to be a clear relationship between the amount of alcohol in alcoholic beverages and the level of this metabolite or some derivates found in saliva (25,29,33). Secondary studies published until now (meta-analyses and systemic reviews) were included in this review (5,34), but to our knowledge, this is the first study reporting not only primary studies assessing oral cancer risk but also the amount of acetaldehyde found in the oral environment. Acetaldehyde has been described as a potential carcinogenic substance (6,7), therefore it could be considered as an indicator of an increasing oral cancer risk. On the other hand, alcohol drinkers have higher levels of this substance in saliva. Hence it would be reliable to find higher levels in subjects who use alcohol-based mouthwash. Kocaelli *et al*. (32) observed a positive relationship in acetaldehyde levels in patients who had oral cancer, as well as in subjects with poor oral health.

This fact enforces the idea that having higher levels of acetaldehyde becomes a risk factor for oral cancer. However, Lachenmeier *et al*. (28) did not find any relationship between alcohol concentration of mouthwash and concentration of acetaldehyde in saliva. This may be due to the variability between subjects and the effect of other factors such as the level of basal acetaldehyde or the microbial oxidation of ethanol (6). Considering this variability and a limited sample (n = 4), a statistical power deficit was observed. Therefore, they published another study with a larger sample in which a relationship was found between the level of acetaldehyde in saliva and the amount of alcohol in mouthwash. Actually, they saw that the higher the alcoholic strength was, the higher the levels of salivary acetaldehyde obtained (29). However, this effect may not last more than 10 minutes, questioning whether alcohol rinsing really is a risk factor for oral cancer (33).

Regarding the risk of oral cancer, there was a great variability in the methodology and results of the included studies. As far as the two meta-analyses included, Gandini *et al*. (5) showed an increasing trend for oral cancer risk due to alcohol-based mouthwash use, but with no statistical significance. On the other hand, Boffetta *et al*. (34) showed a statistically significant higher risk of both oral and oropharyngeal cancer in patients who used mouthwash, but with a very slight effect (OR: 1.01; 95%CI = 1.00 - 1.23 and OR: 1.28; 95%CI = 1.06 – 1.56). However, no statistically significant differences were found between using alcohol-based mouthwash and not using it in non-smoking and/or non-drinking patients (Table 2). This might be explained considering that in the non-stratified comparison, there were also smokers and/or drinkers who might have a higher risk of cancer when they use alcohol-based mouthwash, which increases the overall risk of mouthwash users. Hence smoking or alcohol consumption habits seem to be modifying factors of oral cancer risk for alcohol-based mouthwash use.

On the other hand, Kocaelli *et al*. (32) showed that, although there was a greater presence of acetaldehyde in saliva in subjects who used alcohol-based mouthwash, there were no significant differences between them, but the authors did not stratify on frequency of use.

Considering that the increase of acetaldehyde in saliva lasts for a short period of time (5 – 10 minutes after rinsing), it is necessary to assess whether a high frequency of rinses may increase the risk of oral cancer. Several classic studies indicate that the use of this mouthwash significantly increases the risk of oral cancer (1,2). Guha *et al*. (31) and Ahrens *et al*. (30) obtained similar results, in which subjects who used the alcohol-based mouthwash ≥2 and 3 times/day, respectively, had a significantly higher risk of oral cancer development (OR: 3.54; 95%CI = 2.02 - 6.20; *p*<0.0001 and OR: 3.53; 95%CI = 1.65 - 7.57 respectively). On the contrary, there seemed to be a non-statistically significant predilection in those subjects who used 1 or 2 mouthwashes, with a dose-dependent relationship. Gandini *et al*. (5) did not obtain a statistically significant difference in any of the previous 3 comparisons, but showed a positive dose-dependent trend, assuming great heterogeneity between studies (Table 2). Ahrens *et al*. (30) and Boffetta *et al*. (35) observed that this effect was not significant in non-smokers and non-drinkers, whereas Guha *et al*. (31) argued that there was an increased risk of head and neck cancer regardless of whether the subjects were smokers or drinkers, with mouthwashes with more than 30% of alcohol. Therefore, although the evidence is not entirely clear, it seems that the higher the alcohol strength, the higher the levels of acetaldehyde in saliva increase in the short term (the first minutes) but it obtains baseline values in the medium and long term. However, increasing the frequency of use might lead to higher salivary acetaldehyde and a higher oral cancer risk. Hence, a positive dose-dependent trend is seen in oral cancer risk when using alcohol-based mouthwash (25,29,33).

In patients without other risk factors, the carcinogenic effect seems to be low and still arguable (30-32). On the other hand, there are other risk factors which are clearly established such as smoking or alcohol consumption (30,31,35). The use of an alcohol-based mouthwash with any of the above habits produces an increase in salivary acetaldehyde and a greater carcinogenic effect with statistical significance (31,34). Not only smoking (moderate or heavy smokers) (32) and/or drinking factors (5,6,30,31) increase salivary acetaldehyde, but also other factors such as poor oral health (32), poor oral hygiene (when the number of teeth washing/day is lower, salivary acetaldehyde increases) (30,32). Therefore, these could increase the risk of oral and oropharynx cancer. Even though it is unknown if they increase salivary acetaldehyde, the absence of periodic check-ups to the dentist or complete prothesis users seem to be other risk factors for oral cancer (30). However, these can be intermediate variables (patients with no check-ups to the dentist or complete prostheses users probably have worse oral health and hygiene).

Therefore, it seems that the evidence is limited to the carcinogenic effect of the mouthwash use in patients without other risk factors for oral, oropharynx or head and neck cancer. There might be a trend which indicates the presence of this effect but its existence is still not clear in a meaningful way, and if such an effect existed it would be low.

However, although there were limited results in the included studies due to the heterogeneity between them and the subjects (5,34), it is clear that the risk increases significantly in patients with other risk factors such as smoke and/or alcohol consumption habits.

In conclusion, alcohol-based mouthwash consumption significantly increases salivary acetaldehyde levels in the first few minutes. However, no evidence exists if long-term salivary acetaldehyde levels may increase with a high frequency of mouthwash use. There is still insufficient evidence of whether the use of alcohol-based mouthwash is an independent risk factor for oral or oropharynx cancer. Nonetheless, it does increase the risk when it occurs concomitantly with other risk factors such as smoking or alcohol.
